# Red-crowned crane (*Grus japonensis*) prefers postharvest reed beds during winter period in Yancheng National Nature Reserve

**DOI:** 10.7717/peerj.7682

**Published:** 2019-09-13

**Authors:** Peng Xu, Yalan Zhang, Xiaoran Zhang, Hao Chen, Changhu Lu

**Affiliations:** 1 Nanjing Forestry University, College of Biology and the Environment, Nanjing, Jiangsu, China; 2 Yancheng National Nature Reserve for Rare Birds, Administrative Bureau, Yancheng, Jiangsu, China

**Keywords:** Potential animal food resource, Reed management, Yancheng national nature reserve, Habitat distribution patterns, Red-crowned crane

## Abstract

Reed beds represent an important habitat for the survival of birds by providing favorable foraging and reproduction conditions. Reed management, as a traditional agricultural activity, primarily includes water level control and vegetation removal by cutting. Red-crowned crane (*Grus japonensis*) is one of the most endangered cranes, and their population continues to decline due to habitat loss caused by artificial activities. A lack of research relating to how reed management affects crane habitat distribution patterns throughout the wintering period hinders our ability to offer conservation recommendations. In the present study, we explored the effect of reed management on the habitat distribution patterns and analyzed the food resources of red-crowned crane in the Yancheng National Nature Reserve (YNNR). According to the reed management activities in December, we divided the wintering period into two phases: the preharvest period and the postharvest period. Throughout the wintering period, the number of cranes recorded in the common seepweed (*Suaeda glauca*) tidal flats remained stable, but cranes were rarely recorded in the smooth cordgrass (*Spartina alterniflora*) tidal flats and aquaculture fish ponds. The number of cranes, however, showed a noticeable fluctuation in the reed beds during the two periods. Before the reed harvest, only a small proportion of cranes were recorded in the reed beds (relative abundance = 2.9%). However, more cranes (relative abundance = 61.0%) were recorded after the reed harvest. Water was introduced from adjacent rivers and fish ponds to submerge the cut reed beds. Changes in potential animal food resources (items and biomass) might be one of the vital reasons for the preference of cranes to the postharvest reed beds. Our results suggest that traditional reed management in the YNNR could benefit this flagship crane species that winters in the wetland system. However, as reed harvest has been forbidden in the core zone for conservation purposes since 2016, further research is needed to verify whether forbidding the harvest of reeds is reasonable.

## Introduction

Common reed (*Phragmites australis* (Cav.) Trin. ex Steud.) is a tall grass species that is widely distributed in wetlands and is of economic and ecological importance. Wetlands dominated by the common reed, known as reed beds, occur in both natural conditions and artificial settings that host an array of species of conservation importance (Schmidt et al., 2005; Mer’o et al., 2018). Reed management often occurs by regulating water levels or by harvesting, burning, grazing or mowing either for commercial or conservation purposes (Valkama, Lyytinen & Koricheva, 2008). Many studies have assessed the impact of reed management on passerines. The abundance of passerines decreases in managed reed beds (Mer’o, Lontay & Lengyel, 2015). An important factor that might negatively affect the abundance of passerines in managed sites is food limitation. Important prey groups for passerines, including butterflies (Lepidoptera), beetles (Coleoptera) and some spiders (Araneae), have been reduced in managed sites (Valkama, Lyytinen & Koricheva, 2008). Therefore, managed reed beds are relatively less suitable habitats for passerine species due to the lack of available food resources.

Red-crowned crane (*Grus japonensis*) is considered one of the most endangered cranes in the world (Birdlife International, 2016; Su & Zou, 2012) and continues to face serious threats that affect its population decline. This crane is a wetland specialist, and the Yancheng National Nature Reserve (YNNR) is the largest wintering area for the species in China; It mostly relies upon the core zone of the reserve that has coastal wetlands (Ma, Wang & Tang, 1999). Previous studies have examined the habitat selection of red-crowned cranes under the pressures of wetland loss, degradation and fragmentation and have indicated that habitat transformations due to artificial activities have negatively affected the crane’s distribution, abundance and density (Ma et al., 2006; Liu et al., 2013; Cao et al., 2015). Core zone of the reserve is the most important aggregation area for wintering red-crowned crane. Cranes preferred the common seepweed (*Suaeda glauca*) tidal flats because it had lower vegetation cover and more shallow water, which could provide better foraging habitat for the cranes. They rarely occurred in tidal mudflats dominant by the smooth cordgrass (*Spartina alterniflora*) because of the high smooth cordgrass root density may reduce their foraging efficiency. Due to the high density of reed in uncut reed beds, there was no space for cranes. Reed management, a traditional agricultural activity includes reed harvest and inundation, annually occurs in the core zone of the YNNR in winter. The drainage of reed beds is convenient for reed cutting. Inundation after reed cutting is usually considered necessary for the growth of reeds in the coming spring. However, how such reed management in the core zone affects the crane habitat distribution patterns throughout the wintering period is currently unknown. This knowledge gap hinders our ability to offer conservation recommendations.

In the present study, we (1) explored the habitat distribution patterns of wintering red-crowned cranes in the core zone of the YNNR and especially focused on the influence of reed cutting activity. As the food resources in wintering areas are frequently considered one of the most critical factors affecting the habitat distribution patterns of many bird species (Strong & Sherry, 2000; Wang et al., 2013), we (2) hypothesized that changes in the composition and biomass of potential animal food resources may affect the habitat distribution patterns of red-crowned cranes in areas with reed management.

## Materials & Methods

### Study area

**Figure 1:**
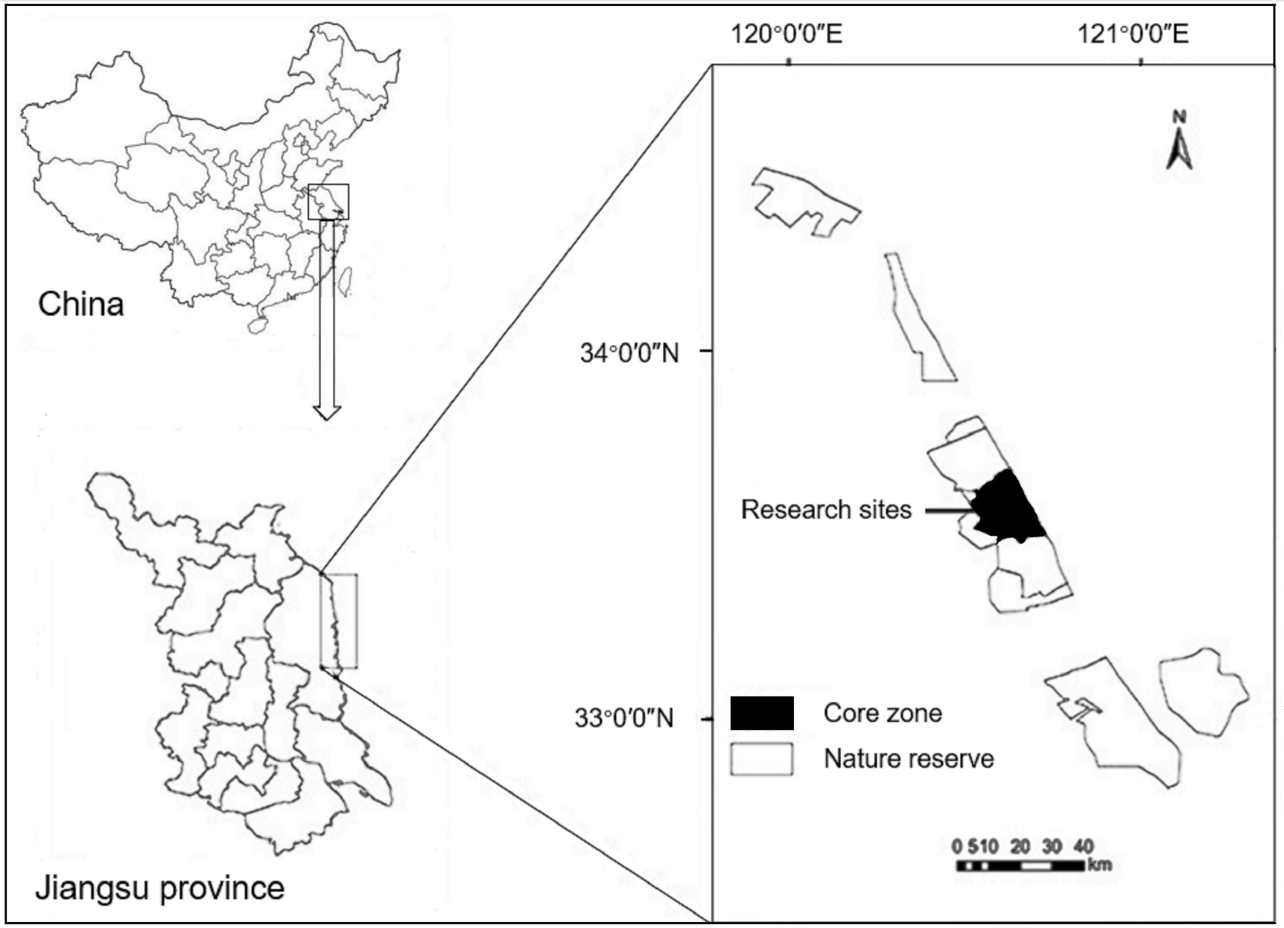
Maps showing the location of the research sites within China and the Yancheng National Nature Reserve.

This study was undertaken in the YNNR (32°48′47″−34°29′28″N, 119°53′45″−121°18′12″E) in Jiangsu Province, eastern China (Fig. 1). Field studies were conducted under the permission from the Administrative Bureau of the YNNR. The YNNR was established in 1983 and was promoted to a national nature reserve in 1992. Currently, the YNNR is the largest coastal zone reserve in China, with a total area of 247,300 ha. As a transition region with a temperate zone, the YNNR has the characteristics of a monsoon marine climate, where the mean temperature is approximately 4 °C in winter, and the annual average precipitation is 980–1,070 mm. Winter precipitation is rare in the area. Vegetation in the core zone of the YNNR shows a clear zonal distribution from the coast to inland with bare tidal flats or supporting communities, such as those dominated by exotic smooth cordgrass, common seepweed, reed and other xeromorphic vegetation (Li et al., 2019). Degree of plant cover is high in reed beds whereas it is relatively low in the common seepweed tidal flats.

### Reed management

As a traditional agricultural activity, local people harvested a partial of the reed beds in the core zone of the YNNR in mid-late December annually. After the reed were harvested, reed beds were exposed, and regulation of shallow water level also occurred by introducing water from adjacent rivers and fish ponds. Thus, we distinguished two phases of the wintering period of red-crowned crane: the preharvest period (8 November to 20 December) and the postharvest period (22 December to 6 March).

### Study species

Red-crowned crane, which is an endemic species in East Asia and is listed as “endangered” on the IUCN Red List of Threatened Species, has a population of approximately 3,000 individuals (Birdlife International, 2016). Its breeding areas include Japan, eastern Russia, Mongolia, and northeastern China. The wintering areas are in Japan, South Korea and the eastern coastal wetlands of China. Annually, red-crowned cranes usually aggregate in large groups before their autumn migratory from northeast China to the YNNR in late October and relatively remained stable until their spring migratory in early March.

### Crane numbers and distribution

**Figure 2:**
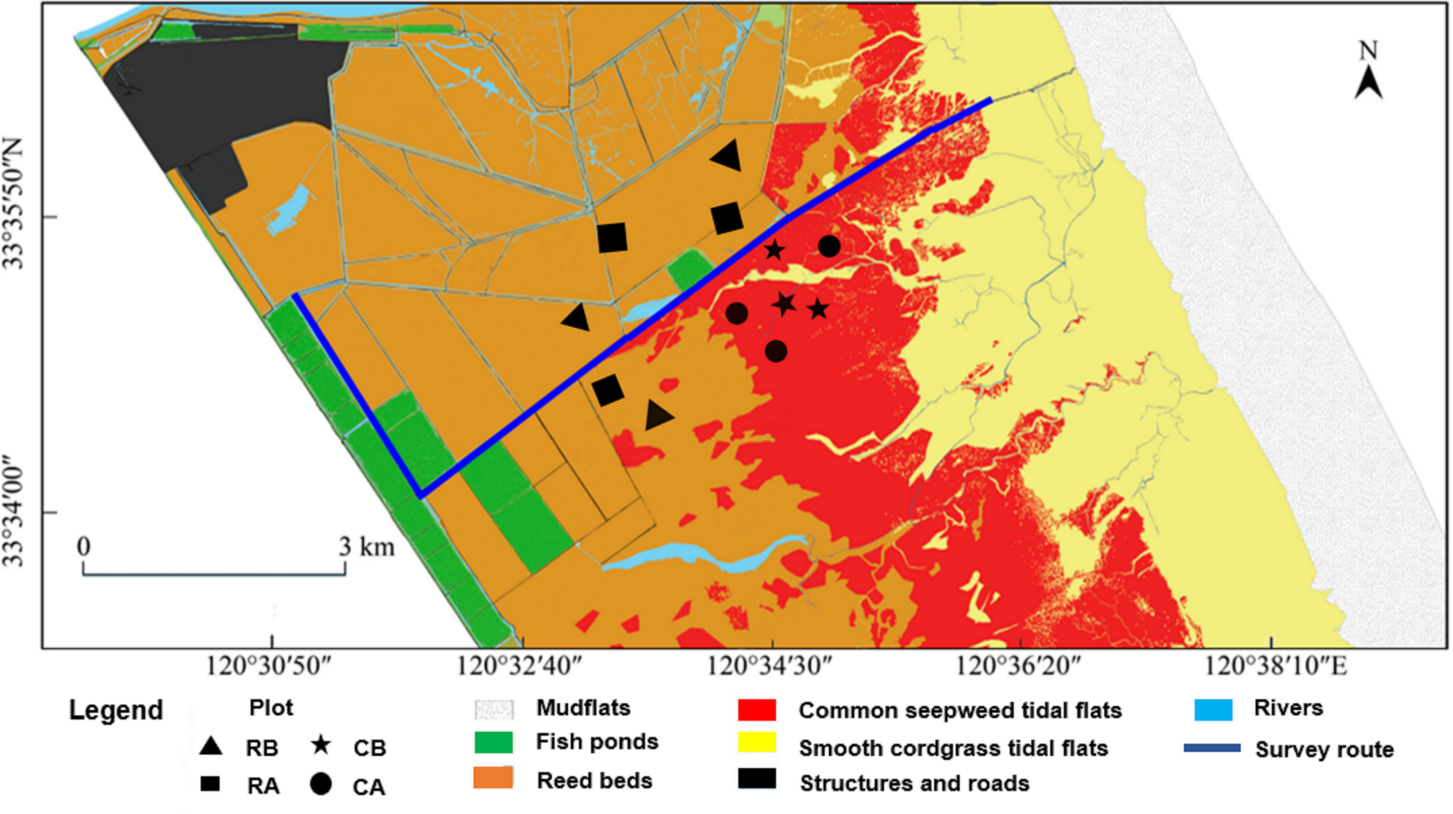
Survey route, habitat types and sampling plots of the research sites in the core zone of the reserve. Plot location: RB, reed beds before reed harvest; RA, reed beds after reed harvest; CB, common seepweed tidal flats before reed harvest; CA common seepweed tidal flats after reed harvest.

Regular crane habitat distribution surveys were performed throughout days with good weather conditions along a fixed route (Fig. 2, approximate nine km) by motorcycle in the core zone from November 2014 to March 2015. Surveys were conducted roughly for every two days, and also for several consecutive days. Because of the crane’s behaviors different though out a day (Shao et al., 2018), the beginning time of our survey was around 9:30 in the morning as wintering cranes were gregarious and shared communal roosting sites at night and usually departed their roosting sites during the morning (06:30–08:00) to forage and returned at night (Lv, 2007). Approximate 2.5 h were spent for each survey. A total of 71 surveys were conducted during the entire winter season. Based on differences in vegetation and micro geomorphic features, the distribution habitat patches of red-crowned crane were divided into four types according to dominant plants, including alien smooth cordgrass tidal flats, common seepweed tidal flats, reed beds and aquaculture fish ponds. Winter fish harvest was common in the buffer and transition zone in the YNNR. However, people in our study area in the core zone did not drain the water off from fish ponds for fish harvesting during the winter. Whenever cranes were encountered, we used a monocular telescope (Nikon, 20 × 60) to scan, count and record the number of birds and habitat types. In order to facilitate the observability, we also get up to scan with greater vision on the mound platforms, which were approximate five m high and built for bird monitoring, along the fixed route. Crane surveys in uncut reedbeds were also conducted before reed harvest. We recorded as “0” when no cranes were observed in uncut reed beds.

### Potential animal food resource collection of the cranes

During the wintering period, the cranes tend to consume animal food resources because of the rapid aging of food plants. In addition, due to the high variability in digestibility of different animal food items, a fecal analysis would create a bias (Redpath et al., 2001). Consequently, field sampling provides a simple, minimally invasive manner for estimating the potential animal food resources available in the foraging habitats of this threatened bird species (Bishop & Li, 2002; Dong et al., 2016). According to previous studies about diet of red-crowned crane by stomach anatomy and fecal analysis, we aggregated the species into four items for the analysis: Annelida, Mollusca (snails and shellfish), Arthropoda (crabs) and Chordata (fish) (Shi & Wu, 1987; Ma, Wang & Tang, 1999). We chose two habitats, including common seepweed tidal flats and reed beds, for specimen collection in the core zone before and after reed management. Three 100 × 100 m plots were marked out in common seepweed tidal flats on the sites most occupied by the cranes and another three were marked out in reed beds before reed harvest. Same sample size was applied after reed cut. We sampled these twelve plots for one time during the study period (Fig. 2). Five random quadrats of one m × one m with 20 cm deep were dug up and removed from within each plot (Jia et al., 2013). To sample crabs in these plots, pitfall trap was deployed in each quadrat by burying cylindrical plastic buckets (20 cm in diameter, 30 cm deep) at the soil surface (Chen et al., 2019). The crabs in each trap were checked in the next day. Because of the shallow water level in these habitats, we directly used a customized fish net (one m × one m, four mm mesh) to sample them before digging. The samples were dried at 60 °C for 48 h. We determined animal species and measured the dry weight using an electronic balance (Shengke Instrument Co., Ltd., Shanghai, China; 2,000 g/0.01 g). Weight determinations were then converted to biomass per plot (g/m^2^).

### Data analysis

We assessed the habitat distribution patterns of red-crowned crane based on its occurrence in different habitats. The relative abundance (%) of cranes in each type of habitats were calculated throughout the winter period. The ratio of the number of cranes that occurred in each habitat type to the total number of cranes in all habitats was estimated as the relative abundance (Zheng et al., 2015) using the following equation:

Relative abundance = Ni/N

“Relative abundance” is the relative number of cranes found in habitat type i over all crane individuals in all habitats; Ni is the recorded number of cranes in habitat i; and N is the total number of cranes in all habitat types. Differences in the relative abundance within or between habitats indicate changes in crane habitat distribution patterns over spatial–temporal scales. Linear regression was used to estimate changes in the crane numbers in different habitats throughout the wintering period. To assess differences in total food biomass between the two phases and the major distribution habitats, we performed a two-way ANOVA with phase/habitat and the interactions as fixed effects followed by a *post hoc* pairwise Tukey’s HSD test (Faraway, 2002). All statistical analyses were performed using the software R (version 3.5.1; R Core Team, 2018).

## Results

### Crane habitat distribution

**Figure 3:**
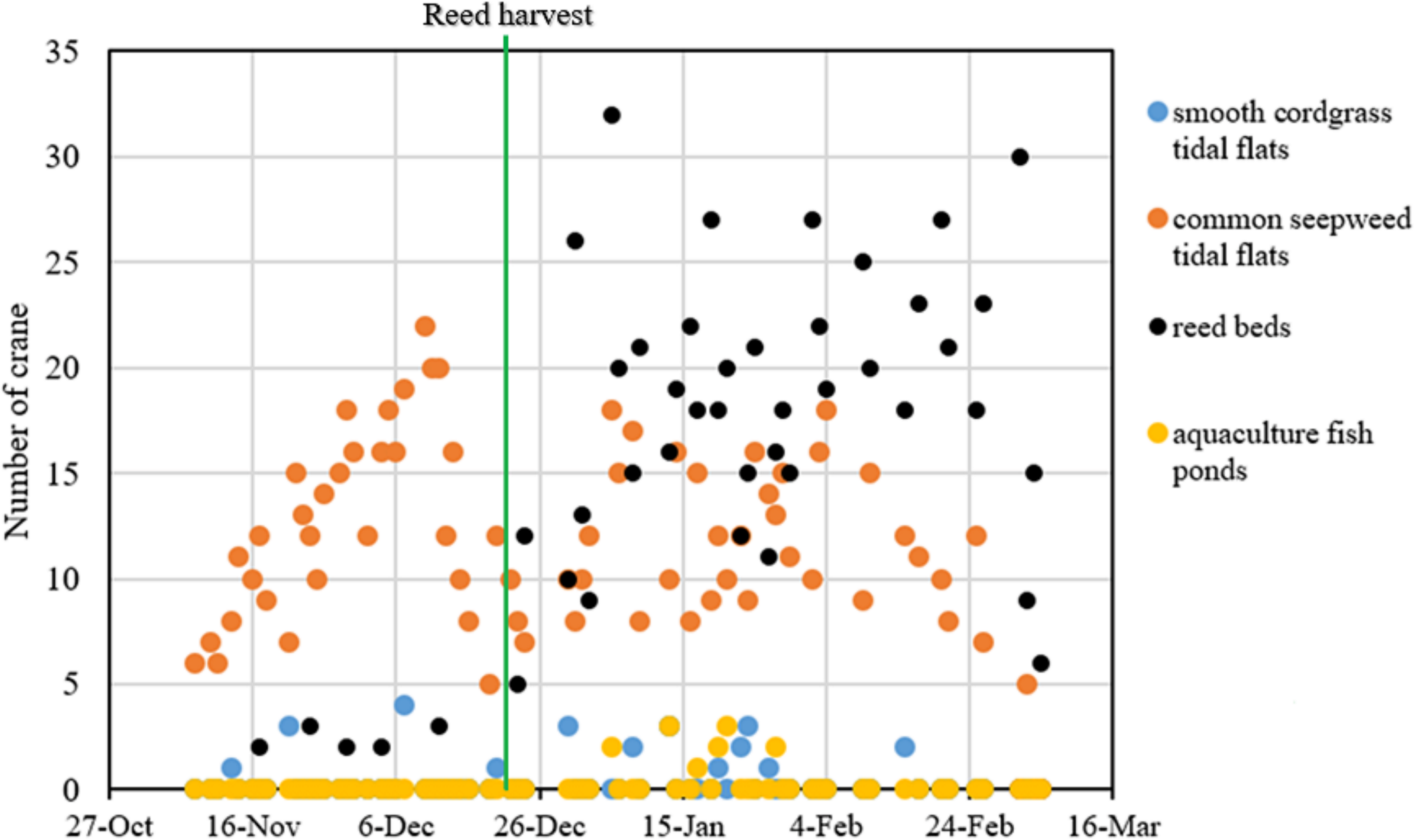
Fluctuation of red-crowned cranes in each habitat throughout the wintering period in the Yancheng National Nature Reserve, China.

From November 2014 to March 2015, we conducted 71 regular red-crowned crane surveys (31 surveys and 40 surveys before and after the reed harvest, respectively). A total of 1,586 individual cranes in the four types of habitat, including smooth cordgrass tidal flats, common seepweed tidal flats, reed beds and aquaculture fish ponds, in the core zone of the YNNR were recorded during our study period. Throughout the wintering period, the number of cranes recorded in the common seepweed tidal flats remained stable (*R*^2^ = 0.031, *F* = 3.244, *df* = 69, and *P* = 0.076, linear regression), whereas the number increased significantly (*R*^2^ = 0.625, *F* = 111.7, *df* = 69, and *P* = 0.000) in the reed beds throughout the wintering period (Fig. 3).

During the study period, the cranes occurred mainly in the common seepweed tidal flats (relative abundance = 51.8%) and reed beds (relative abundance = 45.8%) (Table 1). In addition, the relative abundance and number of cranes were 1.6% and 26 individuals in the smooth cordgrass tidal flats and 0.8% and 13 individuals in the aquaculture fish ponds, respectively, throughout the wintering period, which meant that cranes rarely occurred in these two habitats.

**Figure 4:**
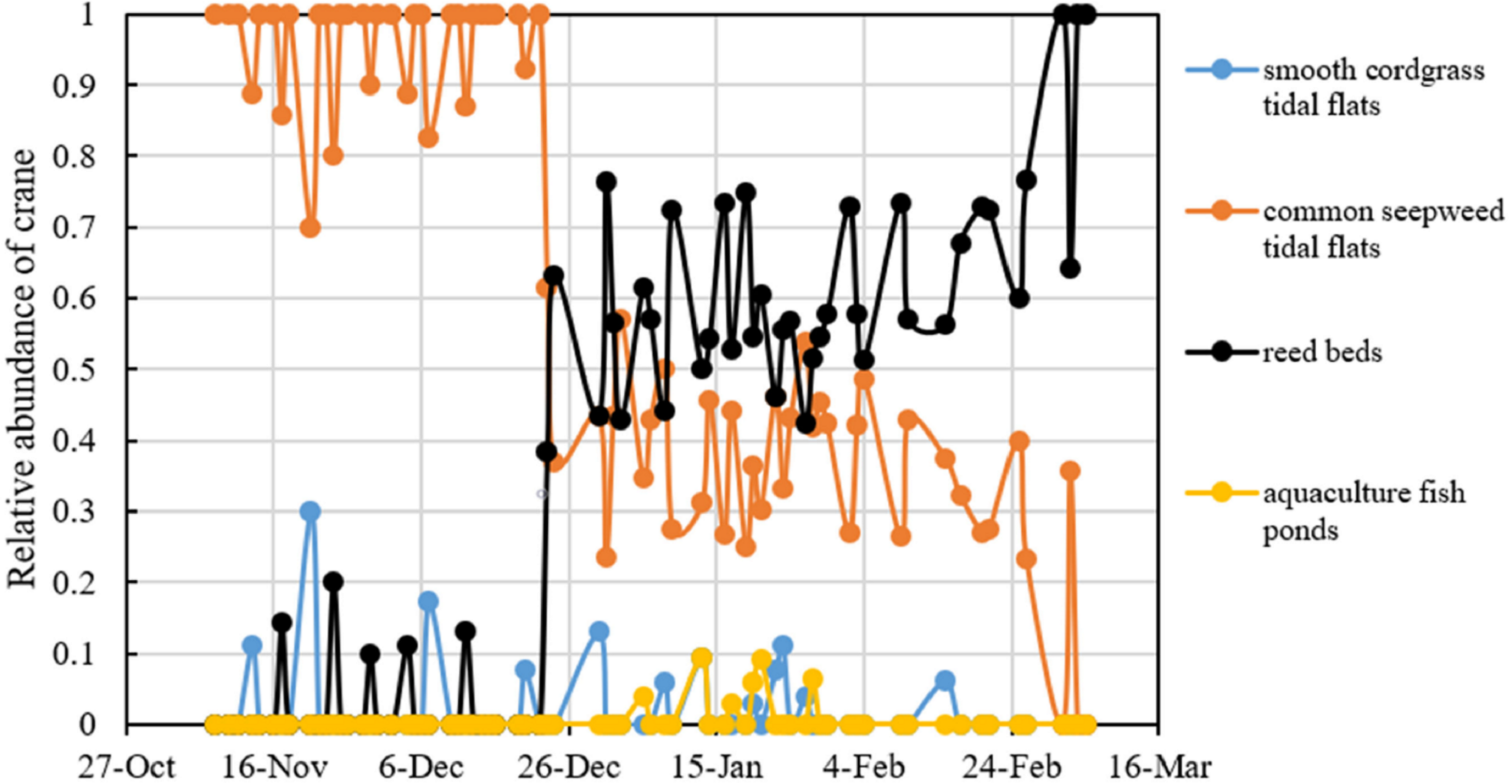
Relative abundance (%) of red-crowned cranes in different habitats of each crane habitat distribution survey in the core zone of the Yancheng National Nature Reserve, China.

Reed management activities noticeably influenced the distribution patterns of red-crowned crane. In the preharvest period, the number of cranes in the common seepweed tidal flats increased significantly (*R*^2^ = 0.176, *F* = 6.19, *df* = 29, and *P* = 0.019), and cranes occurred more often in this habitat (relative abundance = 95.0%) than in the other three habitats (relative abundance = 5.0%) (Table 1). Moreover, cranes were recorded in the common seepweed tidal flats only during 23 surveys (*n* = 31, Figs. 3 and 4). During the postharvest period, both the relative abundance (36.4%) and number of cranes in the common seepweed tidal flats decreased significantly (*R*^2^ = 0.102, *F* = 4.307, *df* = 38, and *P* = 0.045), whereas the relative abundance (61.0%) and number increased and remained stable (*R*^2^ = 0.083, *F* = 3.428, *df* = 38, and *P* = 0.072) in the reed beds until early March before the spring migration (Figs. 3 and 4).

**Table 1:** Relative abundances of red-crowned crane in each habitat type throughout the entire wintering period and in the two phases.

	Smooth cordgrass tidal flats	Common seepweed tidal flats	Reed beds	Aquaculture fish ponds
Entire wintering period	1.6%	51.8%	45.8%	0.8%
Preharvest period	2.1%	95.0%	2.9%	0.0%
Postharvest period	1.5%	36.4%	61.0%	1.1%

### Variability in potential animal food resources

Food biomass was considered the potential animal food resource availability in the common seepweed tidal flats and reed beds. The species composition and biomass of potential animal food resources in these two habitats throughout the two phases contrasted as the relative abundance of red-crowned crane decreased in the common seepweed tidal flats and increased in the reed beds after reed harvest. A total of 14 species were found among these two habitats. More species were recorded in the common seepweed tidal flats than in the reed beds and throughout the wintering period (Table 2). Before the reed harvest, three crab species and only one fish species were sampled in the red beds. However, crab species were not recorded, and the number of fish species increased to seven after the reed harvest due to inundation (Table 2).

**Table 2:** Species composition of the potential animal food resources in the common seepweed tidalflats and reed beds throughout the wintering period in the Yancheng National Nature Reserve, China.

Phylum	No.	Species	Preharvest period		Postharvest period	
			C	R	C	R
Annelid	1	*Tylorrhynchus heterochaetus*	*		*	
Mollusca	2	*Glauconome chinensis*	*		*	
	3	*Cyclina sinensis*	*		*	
	4	*Oncomelania hupensis*	*		*	
Arthropoda	5	*Chiromantes dehaani*	*	*	*	
	6	*Helice tridens tientsinensis*	*	*	*	
	7	*Macrophthalmus japonicus*	*	*	*	
Chordata	8	*Periophthalmus modestus*	*	*	*	*
	9	*Pelteobagrus fulvidraco*				*
	10	*Ilisha elongate*				*
	11	*Erythroculter ilishaeformis*				*
	12	*Carassius auratus*				*
	13	*Mylopharyngodon piceus*				*
	14	*Ophiocephalus argus*				*

**Notes.**

* represents the existence of the species in the habitat.

C common seepweed tidal flats R reed beds

**Figure 5:**
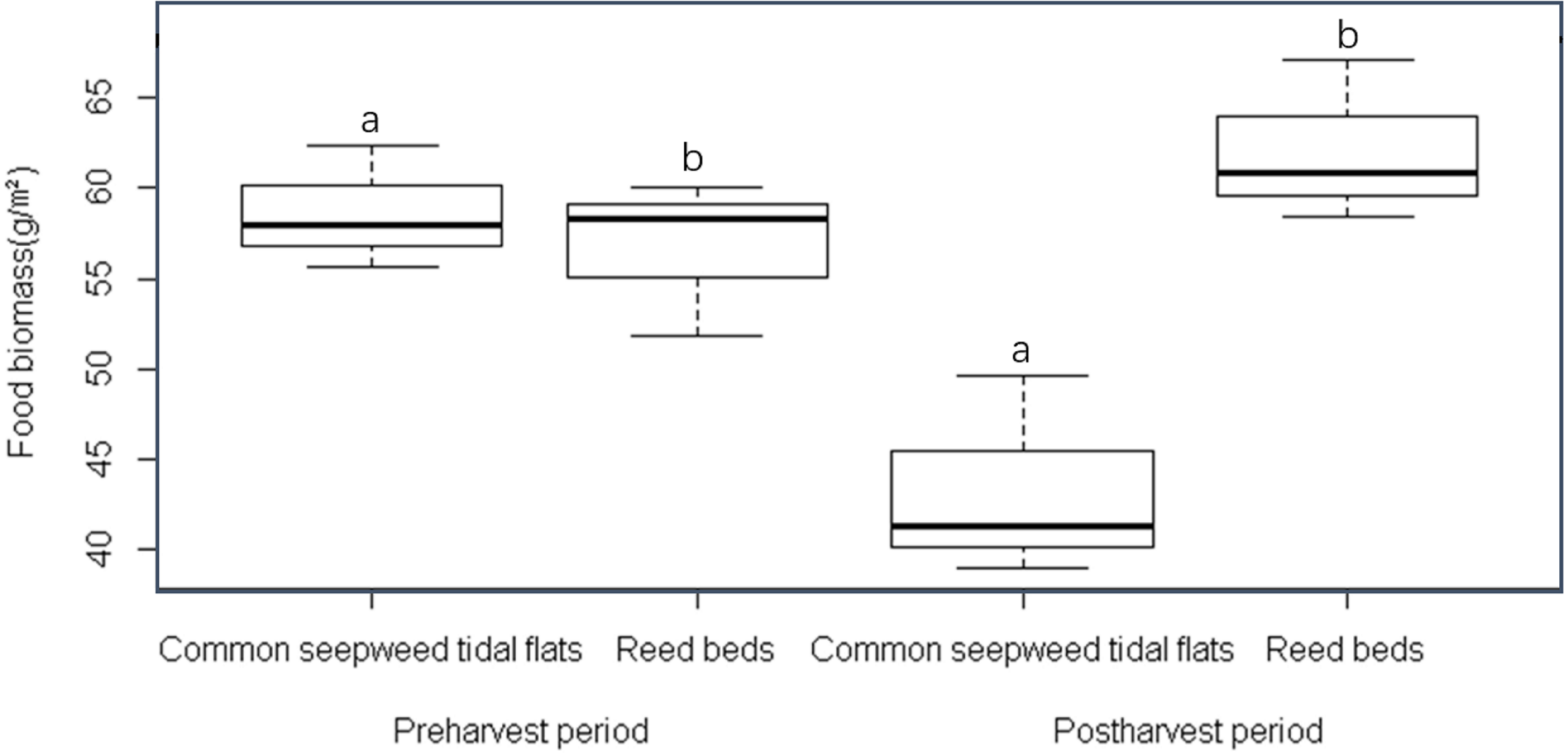
Food variables (g/m^2^) within different habitats and phases in the core zone of the Yancheng National Nature Reserve, China. Different letters (a and b) indicate significant different between habitat types (*P* < 0.05).

It should be noted that the food biomass differed significantly among the habitat types (*n* = 12, *P* = 0.012) due to the interactive effect of phases and habitats (*n* = 12, *P* = 0.004) (Table 3). Food biomass was more abundant in the common seepweed tidal flats than that in the reed beds before the reed harvest, and the food biomass decreased after the reed harvest. In contrast, the food biomass increased in the reed beds after the reed harvest (Fig. 5).

## Discussion

It is essential to understand red-crowned crane distribution patterns to maintain and improve the habitat conditions for this population in the YNNR, which supports the largest population wintering in China. Moreover, with natural wetlands and no human disturbance, the core zone of the reserve is the most important aggregation area for wintering red-crowned cranes (Ma, Wang & Tang, 1999). Red-crowned cranes occurred in four types of habitats, including smooth cordgrass tidal flats, common seepweed tidal flats, reed beds and aquaculture fish ponds, during the wintering period. Similar to the results of previous studies, tidal mudflat habitats dominated by common seepweed were important for wintering red-crowned cranes. Low vegetation cover in common seepweed tidal mudflats are preferred by red-crowned cranes (Liu et al., 2013; Cao et al., 2015). Potential animal food resources were abundant in the common seepweed tidal mudflats. Aquaculture fish ponds with shallow water levels have been reported to be relatively good foraging sites for red-crowned cranes (Ma, Wang & Tang, 1999; Li et al., 2019). However, in the present study, cranes rarely occurred in fish ponds throughout the entire wintering period, potentially because a high-water level impeded the availability of potential food resources. Moreover, people in our study area did not drain the water off from fish ponds during the winter. Cranes also rarely occurred in tidal mudflats dominant by the smooth cordgrass. This may be because the high smooth cordgrass root density hindered the availability of food to cranes, thereby reducing their foraging efficiency (Johnson, 1980; Jason, 2001). Arguably, in the long term, the invasion and expansion of smooth cordgrass has noticeable negative effects on habitation by cranes (Liu et al., 2013; Cao et al., 2015).

**Table 3:** Analysis of variance of the food biomass of red-crowned cranes in relation to reed management and habitat differences (*n* = 12).

Factor	Food biomass	
	*F*	*P*
Phase	3.603	0.094
Habitat	10.500	0.012
Phase*Habitat	15.753	0.004

The crane habitat distribution patterns changed in response to reed management. Before the reed harvest, red-crowned cranes rarely occurred in reed beds in the core zone of the YNNR. Obviously, high density reed and large amount of vegetation cover in this area hindered the occurrence of wintering red-crowned cranes by affecting their movement and foraging. Meanwhile, common seepweed tidal mudflats was proved to be the main habitat for cranes. However, after the reed harvest, cranes occurred more frequently in the reed beds. Reed cutting activities lowered the amount of vegetation cover and the beds were exposed, which may be advantageous to their vigilance and searching for food and drinking water (Ma, Wang & Tang, 1999; Ma et al., 2006; Wang et al., 2011). Food resources are frequently considered as a critical factor affecting crane habitat utilization (Dong et al., 2016). In the present study, water was introduced from adjacent rivers and fish ponds to submerge the reed beds after the reed harvest, which increased the potential animal food resources, such as fish, in the reed beds. The changes in species composition and biomass induced the cranes to forage and inhabit the area. Then, the number of cranes in the reed beds increased significantly after the harvest, and the number of cranes in the common seepweed tidal mudflats decreased, meaning that some individuals moved into postharvest reed beds. Inundation after reed cutting made the reed beds ideal habitats for cranes.

Reed harvest, as a traditional agricultural activity creating high economic value, however, has been forbidden in the core zone of the YNNR since 2016 for conservation purposes. The preservation of reeds may create suitable habitats for breeding passerine birds, such as reed parrotbill (*Paradoxornis heudei*), great reed warbler (*Acrocephalus arundinaceus*) and oriental reed warbler (*A. orientalis*) (Dyrcz & Nagata, 2002; Boulord, Zhang & Wang, 2012; Xiong & Lu, 2013). However, a certain impact was confirmed on the habitat distribution of cranes, including red-crowned crane and common crane (*G. grus*). (Field surveys indicated that common crane also occurred in the reed beds after the reed was cut). The YNNR was established in 1986, and a large area of reed beds exists in the reserve. Reed cutting for economic reasons was common each winter. To date, the habitat distribution patterns of red-crowned cranes were adapted to this agricultural disturbance, and the population wintering in this area reached a peak of more than 1,000 individuals in 1999. In recent years, due to the large-scale fragmentation of coastal habitats, the population was reduced to approximately 400–600 individuals (Lv, 2008; Li et al., 2019). Although the number of red-crowned cranes is declining due to human disturbance, our research indicates that reed harvesting was undoubtedly not a negative cause. Effective and sustainable reed management (harvesting an appropriate area and controlling the shallow water level of reed beds) in the core zone could benefit this flagship crane species that winters in the wetland system of the YNNR. Further studies are needed to estimate whether forbidding the harvest of reeds after 2016 is reasonable.

## Conclusions

Throughout the wintering period, the number of red-crowned cranes that occurred in the reed beds showed a clear fluctuation due to reed management activities in December, and the cranes preferred the postharvest reed beds as their foraging habitat. Our results suggest that traditional reed management in the YNNR could benefit this flagship crane species that winters in the wetland system. Effective and sustainable reed management throughout winter by harvesting appropriate areas and controlling the shallow water level of the reed beds should be considered.

##  Supplemental Information

10.7717/peerj.7682/supp-1 Data S1 Raw data of red-crowned crane numbers and distribution and potential animal food resource collection

## Additional Information and Declarations

### Field Study Permissions

The following information was supplied relating to field study approvals (i.e., approving body and any reference numbers): Field studies were conducted under the permission from the Administrative Bureau of Yancheng National Nature Reserve.

### Data Availability

The following information was supplied regarding data availability: The raw measurements are available in the Supplemental File.
